# *Wickerhamomyces anomalus* Fungemia during Healthcare-Associated Outbreak, Pereira, Colombia, 2025

**DOI:** 10.3201/eid3206.251980

**Published:** 2026-06

**Authors:** Karen M. Ordoñez, Diego H. Caceres, Andres Ceballos-Garzon, Natalia Salazar-Giraldo, Bram Spruijtenburg, Lina M. Velasquez-Orozco, Eelco F.J. Meijer, Gabriel Vinazco, Elaine Cristina Francisco, Rodrigo Oliveira, Patricia Escandon, Jacques F. Meis

**Affiliations:** E.S.E. Hospital Universitario San Jorge de Pereira, Secretaría de Salud de Risaralda, Pereira, Colombia (K.M. Ordoñez, N. Salazar-Giraldo, L.M. Velasquez-Orozco, G. Vinazco); Immuno-Mycologics, Norman, Oklahoma, USA (D.H. Caceres); Universidad del Rosario, Bogota, Colombia (D.H. Caceres, A. Ceballos-Garzon); Radboudumc-CWZ Center of Expertise for Mycology, Nijmegen, the Netherlands (D.H. Caceres, B. Spruijtenburg, E.F.J. Meijer, J.F. Meis); Canisius-Wilhelmina Hospital (CWZ)/Dicoon, Nijmegen (B. Spruijtenburg, E.F.J. Meijer); Universidade Federal de São Paulo, São Paulo, Brazil (E. C. Francisco); Antimicrobial Resistance Institute of São Paulo, São Paulo (E. C. Francisco); Universidade Federal do Paraná, Curitiba, Brazil (E. C. Francisco, J.F. Meis); Bruker Daltonics GmbH & Co. KG, Bremen, Germany (R. Oliveira); Instituto Nacional de Salud, Bogotá (P. Escandon); University of Cologne, Cologne, Germany (J.F. Meis)

**Keywords:** fungi, outbreak, *Wickerhamomyces anomalus*, bloodstream infections, candidemia, Colombia

## Abstract

During March–July 2025, ten cases of *Wickerhamomyces anomalus* bloodstream infection were identified in Pereira, Colombia, mainly affecting pediatric patients; 9 cases occurred in children (8 neonates and a 5-year-old girl) and 1 case was in an adult. Most neonates were preterm and had multiple underlying conditions, such as congenital anomalies, respiratory complications, and adverse perinatal conditions, often compounded by limited prenatal care and maternal infections. All patients required intensive medical interventions, including central and peripheral venous catheters, mechanical ventilation, parenteral nutrition, and, in some cases, surgical procedures. Broad-spectrum antibacterial therapy was widely used, and antifungal treatment, primarily caspofungin, was initiated in half of cases. Antifungal susceptibility testing demonstrated low MICs for all agents. Short tandem repeat genotyping of 6 isolates indicated clonal transmission, supporting a healthcare-associated outbreak. Despite prolonged hospitalizations and severe clinical conditions, all patients survived, highlighting the importance of prompt diagnosis, strict infection control, and appropriate antifungal management.

*Wickerhamomyces anomalus* (synonym *Candida pelliculosa*, *Pichia anomala*, and *Hansenula anomala*) is an uncommon opportunistic yeast increasingly recognized as a cause of healthcare-associated infections, particularly in neonatal and pediatric intensive care units, and is associated with contaminated medical devices and inadequate infection control practices (*1*–*11*). Although the yeast is part of the environmental and sometimes human microbiota, under certain conditions it can cause invasive disease, especially in immunocompromised or critically ill patients (*10*). *W. anomalus* was overrepresented in bloodstream infections (BSIs) among persons who use intravenous drugs and is increasingly recognized as an emerging opportunistic pathogen (*12*).

In recent years, several healthcare-associated outbreaks caused by this yeast have been documented. In 2025, a cluster of *W. anomalus* BSIs was identified in Pereira, Colombia. The affected population consisted predominantly of neonatal and pediatric patients with severe underlying conditions and multiple invasive exposures. We describe the clinical, microbiological, and epidemiologic characteristics of the outbreak, as well as the infection control measures that were implemented and microbiological findings supporting clonal transmission.

## Material and Methods

The outbreak investigation was conducted in accordance with the national guidelines and protocols of the Healthcare-Associated Infections Program of the Instituto Nacional de Salud of Colombia. Epidemiologic investigation, case identification, implementation of infection prevention and control measures, and data collection followed protocols established by the national surveillance system for healthcare-associated infections. This study was conducted as part of a public health outbreak investigation; therefore, institutional review board approval and informed consent were not required.

We collected clinical, epidemiologic, and microbiological data from patients from hospital records and laboratory databases as part of the outbreak investigation and control activities. We defined age groups as follows: neonates (0–30 days of age), infants (31–364 days of age), preschool-aged children (1–5 years of age), school-aged children (6–12 years of age), adolescents (13–17 years of age), and adults (>18 years of age). We extracted data on patient demographics, underlying conditions, exposure to invasive procedures, antimicrobial therapy, and clinical outcomes using a standardized case report form designed for outbreak investigations. We accessed institutional infection prevention records to document infection control measures implemented during the outbreak. We performed descriptive statistical analyses with categorical variables summarized as frequencies and percentages and continuous variables summarized as medians and ranges. All procedures were conducted in accordance with institutional guidelines for outbreak investigation and response.

We processed blood cultures using aerobic and anaerobic BACT/ALERT bottles and incubated them in the BACT/ALERT VIRTUO (bioMérieux, https://www.biomerieux.com) system. We initially identified yeast isolates using the VITEK 2 system with the YST identification card (bioMérieux) and confirmed identification by using matrix-assisted laser desorption/ionization time-of-flight mass spectrometry and molecular sequencing.

We performed antifungal susceptibility testing (AFST) with the VITEK 2 YST AST card (bioMérieux), followed by confirmatory testing with a broth microdilution testing according to the Clinical and Laboratory Standards Institute (CLSI) reference standard M27 (*13*). We prepared fluconazole, voriconazole, anidulafungin, and amphotericin B stock solutions in dimethyl sulfoxide and made subsequent dilutions RPMI 1640 medium. In microdilution plates, we combined 2-fold serial dilutions of antifungals with a yeast inoculum standardized to 1–5 × 10^3^ CFU/mL in a final volume of 200 µL/well. After 24 hours of incubation at 35°C, we determined MICs visually. We defined the MIC endpoints as the lowest drug concentration that produced >50% growth inhibition for the azoles (fluconazole, voriconazole) and anidulafungin and 100% growth inhibition for amphotericin B, compared with the drug-free control well. We ensured quality control using *C. parapsilosis* ATCC 22019 and *C. krusei* ATCC 6258.

For short tandem repeat (SRT) genotyping, we extracted DNA from all cultured isolates with the MagNA Pure 96 instrument and MagNA Pure DNA and Viral NA Small volume kit (Roche, https://www.roche.com) according to the manufacturer’s instructions, as described previously (*14*). We performed multiplex PCR reaction amplifying 6 previously described STR markers with a thermocycler (Analytik Jena, https://www.analytik-jena.us) under identical PCR conditions (*14*). We analyzed amplicons on a 3500 XL genetic analyzer (Thermo Fisher Scientific, https://www.thermofisher.com), determined copy numbers using GeneMapper version 5 software (Thermo Fisher Scientific), and inferred phylogenetic relatedness between isolates using BioNumerics version 7.6.1 (bioMérieux) (*14*).

## Results

### Description of Cases 

**Figure 1:**
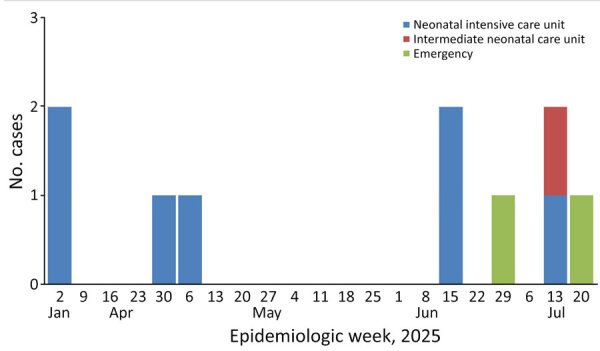
Epidemic curve of bloodstream infections caused by *Wickerhamomyces anomalus* during healthcare-associated outbreak, Pereira, Colombia, March–July 2025.

A total of 10 cases of BSI caused by *W. anomalus* were identified in 1 healthcare institution in Pereira, Risaralda Department, Colombia, during March–July 2025, epidemiologic weeks (EWs) 10–30 (Figure 1). Of the 10 case-patients, 6 were female and 4 were male. Nine cases occurred in pediatric patients, 8 neonates (<30 days of age) and 1 infant (a 5-year-old girl); 1 case was reported in a 65-year-old woman.

Of the 10 patients, 7 were admitted to the neonatal intensive care unit (NICU), 1 was admitted to the intermediate neonatal care unit, and 2 (the infant and the adult patient) were admitted through the emergency department. Two of the 10 case-patients were referred from other healthcare institutions.

The median time from hospital admission to onset of symptoms was 17 (range 6–47) days. Peripheral blood cultures were collected on the same day clinical signs and symptoms first appeared. The median interval from blood culture collection to the report of a positive result was 5 (range 2–14) days. The median time from hospital admission to microbiological confirmation of infection was 27 (range 11–50) days.

Initial identification of the isolates was performed using the automated VITEK 2 system, which identified the organisms as *Candida pelliculosa* (currently classified as *W. anomalus*). Because of the uncommon nature of this identification, all isolates were reported to the central laboratory of the State Department of Health of Risaralda, then referred to the National Institute of Health, according to the national guidelines for submission of compulsory notification of mycosis (https://www.ins.gov.co/BibliotecaDigital/criterios-para-el-envio-de-aislamientos-bacterianos-y-levaduras-genero-candida-spp-recuperados-en-iaas-para-confirmacion-de-mecanismos-de-resistencia-2024.pdf). Isolates were also referred for confirmatory testing using matrix-assisted laser desorption/ionization time-of-flight mass spectrometry (Bruker, https://www.bruker.com) and molecular analysis, as well as antifungal susceptibility testing. Those procedures were conducted by the Grupo de Microbiologia at the Instituto Nacional de Salud (Bogotá, Colombia); the Studies in Translational Microbiology and Emerging Diseases Research Group, School of Medicine and Health Sciences, Universidad del Rosario (Bogotá, Colombia); the Division of Infectious Diseases, Escola Paulista de Medicina–Universidade Federal de São Paulo (São Paulo, Brazil); and the Department of Medical Microbiology and Immunology, Canisius-Wilhelmina Hospital/Dicoon (Nijmegen, the Netherlands).

AFST results obtained with the VITEK 2 YST AST card (bioMérieux) were concordant with the reference broth microdilution method performed according to CLSI (*13*). Both methods confirmed that *W. anomalus* isolates exhibited low MIC values for all antifungal agents tested. The reference CLSI method yielded the following results: the MIC range (and mode) for fluconazole was 2–4 µg/mL (4 µg/mL), for itraconazole was 0.06–0.25 µg/mL (0.12 µg/mL), and for amphotericin B was 0.25–1 µg/mL (0.5 µg/mL). All isolates had an anidulafungin MIC of <0.03 µg/mL. Given the absence of both clinical breakpoints and epidemiologic cutoff values for *W. anomalus* from CLSI, no formal interpretation (e.g., susceptible, resistant, or non–wild-type) can be assigned to those results.

**Figure 2:**
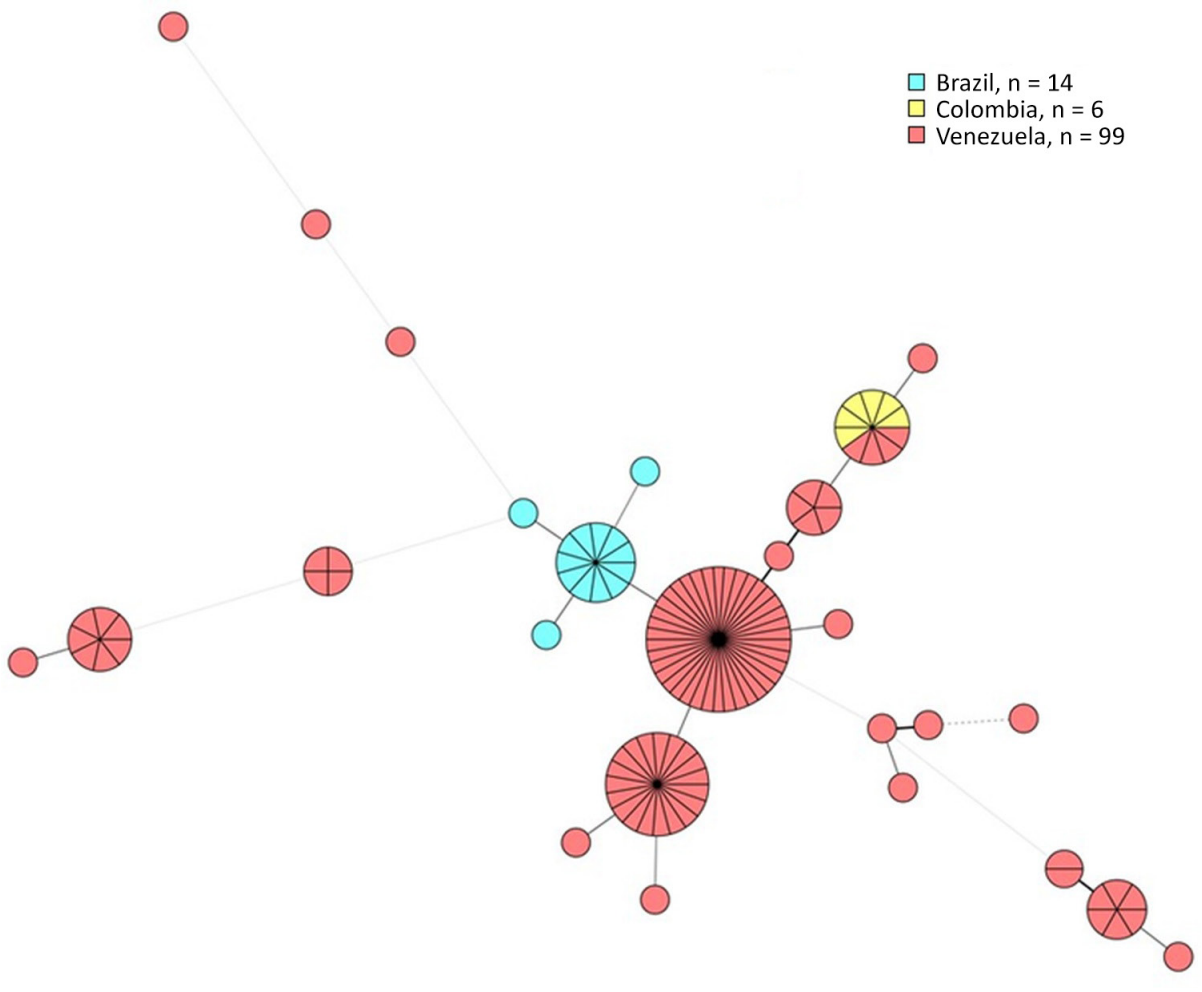
Minimum-spanning tree of *Wickerhamomyces anomalus* genotypes based on short tandem repeat genotyping in study of *W. anomalus* fungemia during healthcare-associated outbreak, Pereira, Colombia, 2025. Isolates from Colombia are compared with control isolates from Venezuela and Brazil (*1*,*15*).

Of 10 available isolates, 6 (cases 5–10) were subjected to genotypic analysis (Table 1). To assess genetic diversity and potential transmission patterns, we performed STR genotyping and compared our results to control outbreak isolates from Venezuela and Brazil. The STR profiles indicated clonal transmission, because all 6 isolates exhibited an identical genotype suggesting a common source or closely linked transmission chain (Figure 2).

**Table 1:** Summary of comorbidities and intrinsic risk factors of patients with *Wickerhamomyces anomalus* fungemia during healthcare-associated outbreak, Pereira, Colombia, 2025

Case	Underlying conditions	Intrinsic risk factors
1	Esophageal atresia	Age 15 d. Cesarean delivery, transverse fetal position, neonatal hypotonia. Referred from another hospital
2	Trisomy 21	Age 10 d. Poor neonatal adaptation, Cesarean delivery
3	Fetal growth restriction, polyhydramnios	Age 18 d. Prematurity (37.5 wks), suspected tracheoesophageal fistula
4	Neonatal hypoxia, respiratory distress	Age 30 d. No prenatal care, meconium-stained fluid, maternal infection. Referred from another hospital
5	Respiratory distress syndrome	Age 14 d. Prematurity (32 wks), rupture of membranes, gastrointestinal bleeding
6	Hyaline membrane disease, suspected congenital pneumonia	Age 30 d. Extreme prematurity (28.5 wks), high septic risk
7	Suspected neonatal sepsis	Age 5 y. Fever, leukocytosis, abdominal discomfort
8	Respiratory failure, apnea	Age 30 d. Prematurity (31.5 wks), twin pregnancy, growth restriction
9	Upper gastrointestinal bleeding (adult case)	Age 63 y. Prior hospitalization, incomplete diagnostic workup
10	Prematurity (34.1 weeks)	Age 30 d. Twin pregnancy, no prenatal care

### Summary of Underlying Conditions and Intrinsic Risk Factors 

The analyzed cases primarily involved neonates with multiple underlying conditions and intrinsic risk factors for candidemia. Congenital anomalies were common, including esophageal atresia, more occasionally tracheosophageal fistula, and suspected trisomy 21. Prematurity was reported in 3 patients; gestational age ranged from 28.5 to 37.5 weeks. Prematurity was frequently accompanied by severe respiratory complications, such as respiratory distress syndrome, hyaline membrane disease, apnea, and requirement for intubation and mechanical ventilation. Additional perinatal complications included fetal growth restriction and twin pregnancies, which both contributed to increased neonatal vulnerability (Table 1).

Maternal factors also played a role. Absence of prenatal care, untreated vaginal infections, premature rupture of membranes, and meconium-stained amniotic fluid were recurrent findings. Those conditions were associated with neonatal hypoxia, hypotonia, low Apgar scores, and episodes of hypoglycemia. Several neonates were at high risk for sepsis, frequently linked to maternal fever, leukocytosis, and suspected congenital pneumonia. An isolated case of upper gastrointestinal bleeding was documented in an adult patient, unrelated to the neonatal population (Table 1). Overall, the main predisposing factors identified were prematurity, congenital malformations, respiratory complications, and adverse maternal conditions, underscoring the importance of comprehensive perinatal management and close clinical monitoring from birth.

### Common Exposure Factors 

Across the analyzed cases, several invasive procedures and therapeutic interventions were consistently identified. The most frequent exposure factors included epicutaneous and central venous catheters, as well as umbilical arterial and venous lines, which were often maintained for extended periods. Endotracheal mechanical ventilation was common, particularly among neonates with respiratory distress. Parenteral nutrition was widely administered (n = 9), reflecting the presence of gastrointestinal complications and the need for postoperative nutritional support (Table 2). Multiple surgical procedures were performed, including laparotomy, intestinal resection and anastomosis, correction of malrotation and duodenal atresia, colostomy or ileostomy, and peritoneal lavage, frequently accompanied by abdominal drainage or the use of negative pressure therapy devices. Endoscopic procedures such as esophagogastroduodenoscopy were also reported (Table 2). Overall, these findings highlight the high frequency of invasive device use, complex surgical interventions, parenteral nutrition, and broad antimicrobial exposure, factors that collectively predispose patients to nosocomial infection and associated complications.

**Table 2:** Common exposure factors identified in cases of *Wickerhamomyces anomalus* fungemia during healthcare-associated outbreak, Pereira, Colombia, 2025

Exposure factor	Frequency, no. (%) n = 10	Clinical context
Epicutaneous catheter	8 cases (80)	Long-term intravenous therapy, parenteral nutrition
Peripheral catheter	9 cases (90)	Initial intravenous access, supportive therapy
Central venous catheter	3 cases (30)	Advanced monitoring, multiple drug infusions
Umbilical venous/arterial catheter	1 case (10)	Neonatal stabilization
Endotracheal tube	8 cases (80)	Airway management, mechanical ventilation
Nasofibrolaryngoscopy	1 case (10)	Diagnostic airway evaluation
Thoracostomy tube	1 case (10)	Pleural drainage postsurgery
Femoral catheter	1 case (10)	Complex surgical/postoperative care
Jugular catheter	1 case (10)	Additional vascular access
Parenteral nutrition	9 cases (90)	Nutritional support in gastrointestinal surgery or prematurity
Major abdominal surgery	6 cases (60)	Laparotomy, intestinal resection, anastomosis
Antimicrobial therapy	10 cases (100)	Empiric and targeted treatment for infection risk

### Invasive Devices and Surgical Interventions 

**Figure 3:**
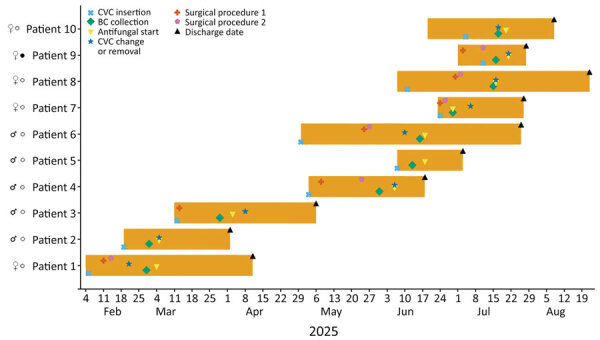
Timeline of clinical events during outbreak of *Wickerhamomyces anomalus* fungemia in Pereira, Colombia, March–July 2025. Each bar represents the hospitalization period of each affected patient; symbols indicate key clinical events. Male patients are indicated with ♂, female patients with ♀, pediatric patients with white circle, adult patient with a filled circle. BC, blood culture; CVC, central venous catheter.

Invasive device use was a predominant feature among all analyzed cases, reflecting the complexity of care required for critically ill neonatal and pediatric patients. Peripheral and epicutaneous catheters were the most frequently used devices; peripheral catheters were documented in 9 (90%) cases and epicutaneous catheters were documented in 8 (80%) cases, primarily for prolonged intravenous therapy and parenteral nutrition. Endotracheal tubes were also placed in 8 (80%) cases and were most commonly associated with mechanical ventilation during surgical procedures or respiratory distress management. Central venous catheters were inserted in 3 (30%) patients, requiring advanced hemodynamic monitoring or administration of multiple drugs, whereas umbilical venous and arterial catheters were used in 1 neonate (10%) during early stabilization. Other less frequently reported devices included femoral and jugular catheters and thoracostomy tubes (each reported 1 case [10%]), generally related to complex postoperative care (Table 2; Figure 3).

Device exposure was often simultaneous and prolonged; epicutaneous or peripheral catheters used in combination with endotracheal tubes were the most prevalent. The cumulative exposure to multiple invasive devices, coupled with extended parenteral nutrition and sequential broad-spectrum antimicrobial therapy, highlights the increased vulnerability of this population to healthcare-associated infections. Extensive surgical interventions were also documented, particularly in neonates with congenital or gastrointestinal anomalies. In 1 complex case, multiple abdominal surgeries were required to manage congenital malformations and postoperative complications, culminating in esophageal reconstruction with gastric interposition. The timeline of interventions and identified risk factors illustrates the temporal overlap of hospitalizations, clinical procedures, and key events such as central venous catheter manipulation and antifungal therapy initiation, which together highlight potential periods of common exposure and support the hypothesis of healthcare-associated transmission (Figure 3).

### Summary of Antimicrobial Therapy Use 

According to hospital protocol, antibiotic therapy in neonates was initiated upon the diagnosis of sepsis; all neonates received antibiotics per protocol. In adults, antibiotic therapy was initiated only in patients with documented bacterial coinfection. Antimicrobial exposure was extensive across all cases; multiple sequential and combination regimens. β-lactams were the most frequently used class in all cases. Among those, ampicillin was administered in 6 cases (60%), oxacillin was administered in 4 cases (40%), and cefepime was administered in 4 cases (40%). Cefotaxime and ampicillin/sulbactam were each used in 2 cases (20%), whereas piperacillin/tazobactam, meropenem, and ertapenem were documented in 3 cases (30%) (Table 3). Aminoglycosides were highly prevalent; gentamicin was used in 7 cases (70%) and amikacin was used in 6 cases (60%). Vancomycin was administered in 6 cases (60%), primarily for gram-positive coverage. Metronidazole was included in 6 cases (60%) for anaerobic coverage in abdominal surgical scenarios (Table 3).

**Table 3:** Summary of antimicrobial drugs used for patients with *Wickerhamomyces anomalus* fungemia during healthcare-associated outbreak, Pereira, Colombia, 2025

Antimicrobial drug	Average duration	No. (%) cases
Ampicillin and gentamicin	2–8 d	7 (70)
Amikacin	1–10 d	6 (60)
Vancomycin	2–26 d	6 (60)
Metronidazole	2–15 d	6 (60)
Oxacillin	2–8 d	4 (40)
Cefepime	3–36 d	4 (40)
Piperacillin/tazobactam	2–3 d	3 (30)
Meropenem	4–5 d	3 (30)
Ertapenem	4 d	2 (20)
Cefotaxime	6 d	2 (20)
Ampicillin/sulbactam	5 d	2 (20)
Caspofungin	14 d	10 (100)
Fluconazole*	6 d	1 (10)

*Switched from caspofungin.

Antifungal therapy (caspofungin) was initiated in all cases; in 1 case, fluconazole was documented after a switch from caspofungin after 7 days of treatment, and fluconazole was administered for an additional 6 days. The average duration of caspofungin therapy was 14 days (range 7–22 days) (Table 3).

Overall, antimicrobial regimens were characterized by frequent changes and combination therapy. Those factors reflect the complexity of infections and surgical interventions in critically ill neonates and pediatric patients.

### Containment and Infection Control Measures 

After the detection of fungal BSIs, a series of infection control interventions were implemented and reinforced. Environmental disinfection with sodium dichloroisocyanurate was intensified, progressively increasing the chlorine concentration in NICUs and PICUs to 4,000 ppm. Cleaning and disinfection routines were performed >2×/day; deep terminal cleaning was conducted weekly. For patients who had invasive devices, bathing using chlorhexidine (2%) was standardized to 3×/week and later increased to daily practice. Hygiene audits and staff training sessions were conducted weekly to reinforce hand hygiene and aseptic techniques. Environmental surveillance, including surface cultures from critical areas such as parenteral nutrition preparation rooms and air circulation systems, was performed biweekly to assess microbial contamination and guide interventions. Following the hospital infection control manual, surveillance cultures were obtained from all 8 neonatal incubators. Specimens were collected from internal surfaces where infants were placed and from the drainage systems that remove the water generated by incubators. In addition, environmental cultures were obtained from operating rooms (6 samples). All cultures yielded negative results.

### Outcomes 

All patients had negative follow-up blood cultures at 72 hours after initiation of antifungal therapy. The median length of hospital stay was 44 days, and all patients were discharged alive.

## Discussion

This outbreak of 10 bloodstream infections caused by *W. anomalus* highlights the vulnerability of critically ill neonates and pediatric patients to emerging opportunistic fungal pathogens. Although several outbreaks have been reported over the years (Table 4), few studies have used robust molecular genotyping for confirmation. We used a recently developed panel of 6 highly polymorphic microsatellite markers, validated with whole-genome sequencing single-nucleotide polymorphism calling, to detect a fungemia outbreak of *W. anomalus* in a single hospital in Colombia and compared results with previously published outbreaks in Venezuela and Brazil (*1*,*20*). All Colombia isolates displayed an identical genotype, and the STR assay is known to have a high discriminatory power, strongly suggesting clonal transmission, which is supported by the epidemiologic data. Nonetheless, WGS is needed for confirmation of the nosocomial transmission (*20*). Curiously, the Colombia isolates clustered with the Venezuela isolates; some even displayed identical genotypes. That clustering indicates transmission between those countries, which could be encouraged by patient transfer. Alternatively, a population of *W. anomalus* could have spread within the environments of Colombia and Venezuela recently, after which the patients acquired the yeast from there.

**Table 4:** Summary of reported *Wickerhamomyces anomalus* (synonyms *Candida pelliculosa*, *Pichia anomala*, and *Hansenula anomala*) outbreaks worldwide*

Reference	Year	Country	Key findings
(*10*)	2021	China	Case–control study; broad-spectrum antibiotics and prolonged hospital stay increased risk; biofilm formation associated. No deaths.
(*15*)	2009–2021	China	307 isolates; high resistance to fluconazole (48.5%) and voriconazole (34.5%) (broth microdilution, CLSI methods M27 [*13*]); 118 genotypes; nosocomial outbreaks identified.
(*14*)	2018–2019	India	New STR scheme (6 markers); 90 isolates; 38 genotypes; 4 simultaneous outbreaks within a single hospital.
(*11*)	2015	South Korea	11 patients; multiple risk factors; effective outbreak control after environmental interventions. No deaths.
(*16*)	2014–2016	China	13 cases; 38% mortality; risk factors include ventral venous catheter, antibiotics, TPN; high azole MICs (CLSI and EUCAST).
(*5*)	2013	Brazil	Neonatal outbreak; possible clonal origin; uncommon pathogen.
(*6*)	2013	China	Neonatal outbreak; 6 cases, 1 death; single predominant clone; control achieved with prophylaxis and hygiene measures.
(*9*)	2012–2013	China	14 neonates infected; 2 distinct clones; good clinical outcomes; 2 NICU outbreaks. High in vitro susceptibility to multiple antifungals (susceptibility testing method not described). No deaths.
(*1*)	2002–2004	Brazil	Outbreak affecting 17 children with 41% mortality, linked to central venous catheters; caused by a single strain.
(*17*)	2001	Croatia	*H. anomala* infection in 8 ICU adults. Case-control analysis (32 patients) identified blood alkalosis duration as the only significant risk factor. Source was not identified.
(*7*)	1998	Brazil	Four infants with *P. anomala* infection, one of whom also had *Candida parapsilosis* infection. *P. anomala* acquisition was likely exogenous.
(*18*)	1997	Brazil	24 cases outbreak, in an oncological hospital in Rio de Janeiro. Leukemia was reported in 65% of cases, demonstrating the opportunistic behavior of *H. anomala.*
(*8*)	1996–1998	India	379 children/neonates affected; transmission through healthcare workers’ hands; control achieved through strict hygiene.
(*19*)	1986	United Kingdom	52 colonized neonates, 8 developed infections: 5 fungaemia, 2 fungaemia with ventriculitis, 1 ventriculitis. Seven were very low birthweight (<1,500 g). All were successfully treated with 5-flucytosine and amphotericin B.

*CLSI, Clinical Laboratory Standards Institute; EUCAST, European Committee on Antimicrobial Susceptibility Testing; NICU, neonatal intensive care unit; STR, short tandem repeat; TPN, total parenteral nutrition.

The predominance of cases in neonates, particularly preterm infants with multiple underlying conditions, underscores the interplay of intrinsic risk factors, such as prematurity, congenital anomalies, and severe respiratory compromise, with extrinsic exposures, such as invasive devices, parenteral nutrition, and extensive surgical interventions (*2*,*3*,*7*). Maternal factors, including limited prenatal care and perinatal complications, further contributed to neonatal susceptibility. The median intervals from hospital admission to symptom onset (17 days) and to microbiological confirmation (27 days) emphasize the diagnostic challenges associated with rare yeasts. Antifungal susceptibility testing revealed that timely initiation of targeted therapy, primarily with echinocandins, can be critical in managing such infections. However, prophylactic strategies and empiric antifungal therapy remain complex decisions in high-risk neonatal populations.

The clinical interpretation of the MIC values is challenging because of the absence of species-specific clinical breakpoints for *W. anomalus* from the CLSI, the methodology used in this study. To provide context for our findings, we referenced the epidemiologic cutoff values (ECOFFs) established by EUCAST (*21*). According to EUCAST ECOFFs (fluconazole >32 µg/mL, anidulafungin >0.12 µg/mL, amphotericin B >2 µg/mL), all isolates in our cohort would be classified as wild-type, exhibiting no acquired resistance mechanisms. Crucially, however, ECOFFs are method-specific, and applying EUCAST criteria to CLSI-derived data is for informational purposes only and does not constitute a formal interpretation. Consequently, although the low MICs suggest that the circulating clone is likely drug-susceptible, the definitive correlation of those in vitro results remains uncertain. The *W. anomalus* isolates analyzed in this study demonstrated markedly higher susceptibility to all antifungal agents tested. That finding contrasts with several reports from Asia, in which isolates frequently exhibited elevated MICs, particularly to azoles such as fluconazole and voriconazole. Recent studies from China and India have documented substantial high non–wild-type rates and high azole MICs, suggesting regional variability in antifungal susceptibility patterns. India reported that 50/70 (72%) *W. anomalus* isolates were non–wild-type for fluconazole, 17% demonstrated cross-resistance with voriconazole, and 1.4% demonstrated cross-resistance with micafungin (*22*). A larger, similar study from China found 149/307 (48.5%) non–wild-type to fluconazole and 106/307 (34.5%) voriconazole (*15*).

A pan–azole-resistant and pan–echinocandin-resistant *W. anomalus* bloodstream isolate was recently reported in China (*23*). Although most isolates remain susceptible, the emergence of multidrug resistance represents a growing threat to clinical management, particularly in resource-limited settings (*23*).

This outbreak was closely associated with exposure to multiple invasive devices, prolonged hospitalization, parenteral nutrition, and broad-spectrum antimicrobial use, reflecting a classic pattern of healthcare-associated infections in intensive care settings (*2*,*3*,*7*). Infection control interventions, such as enhanced environmental cleaning, standardized chlorhexidine bathing, and staff education on aseptic techniques, were instrumental in containing the outbreak, illustrating the importance of rapid, multifaceted infection prevention strategies in high-risk units. A search for the environmental origin of the outbreak was conducted, but *W. anomalus* was not detected after testing numerous inanimate surfaces. Although the source was not uncovered, implementing strict infection control measures after the outbreak was recognized was successful in curbing ongoing cases of fungemia.

Despite the severity of underlying conditions and the complexity of care, all patients survived, suggesting that early recognition, appropriate microbiological work-up, adherence to strict infection control protocols, and timely initiation of antifungal therapy are pivotal in favorable outcomes. This series reinforces the need for vigilance regarding uncommon yeasts in neonatal and pediatric intensive care units, particularly in regions where surveillance is limited.

The first limitation of this study is that the small number of cases and absence of a comparison group limited our ability to assess independent risk factors or establish causal associations. Despite extensive environmental sampling, the source of the outbreak could not be identified. Interpretation of antifungal susceptibility results was constrained by the lack of species-specific breakpoints for *W. anomalus*. In addition, the single-center, retrospective design might limit generalizability and introduce incomplete data capture.

In conclusion, *W. anomalus* BSI represents a rare but clinically significant nosocomial infection, predominantly affecting preterm and critically ill neonates with multiple underlying conditions and extensive exposure to invasive procedures. Comprehensive perinatal management, strict aseptic care, and robust infection control measures are essential to prevent outbreaks and optimize patient outcomes. Ongoing surveillance and reporting are critical to improving understanding of epidemiology, risk factors, and effective management of this emerging pathogen.
